# First dose in children: physiological insights into pharmacokinetic scaling approaches and their implications in paediatric drug development

**DOI:** 10.1007/s10928-012-9241-9

**Published:** 2012-02-05

**Authors:** Ashley Strougo, Thomas Eissing, Ashraf Yassen, Stefan Willmann, Meindert Danhof, Jan Freijer

**Affiliations:** 1 Division of Pharmacology, Leiden/Amsterdam Center for Drug Research, Leiden University, Einsteinweg, 55, P.O. Box 9502, Leiden, The Netherlands; 2 Global Clinical Pharmacology & Exploratory Development, Astellas Pharma Global Development Europe, Elisabethhof, 1, P.O. Box 108, 2350 AC Leiderdorp, The Netherlands; 3 Systems Biology & Computational Solutions, Bayer Technology Services GmbH, Building 9115, 51368 Leverkusen, Germany; 4 Centre for Human Drug Research, Zernikedreef 10, 2333 CL Leiden, The Netherlands

**Keywords:** Allometric scaling, Physiologically-based pharmacokinetic models, Pediatrics, Bridging, Scaling, Extrapolation

## Abstract

Dose selection for “first in children” trials often relies on scaling of the pharmacokinetics from adults to children. Commonly used approaches are physiologically-based pharmacokinetic modeling (PBPK) and allometric scaling (AS) in combination with maturation of clearance for early life. In this investigation, a comparison of the two approaches was performed to provide insight into the physiological meaning of AS maturation functions and their interchangeability. The analysis focused on the AS maturation functions established using paracetamol and morphine paediatric data after intravenous administration. First, the estimated AS maturation functions were compared with the maturation functions of the liver enzymes as used in the PBPK models. Second, absolute clearance predictions using AS in combination with maturation functions were compared to PBPK predictions for hypothetical drugs with different pharmacokinetic properties. The results of this investigation showed that AS maturation functions do not solely represent ontogeny of enzyme activity, but aggregate multiple pharmacokinetic properties, as for example extraction ratio and lipophilicity (log P). Especially in children younger than 1 year, predictions using AS in combination with maturation functions and PBPK were not interchangeable. This highlights the necessity of investigating methodological uncertainty to allow a proper estimation of the “first dose in children” and assessment of its risk and benefits.

## Introduction

Dose selection for “first in children” trials of new pharmaceuticals is often based on scaling of data from adults to children. The type of data that should be scaled is dependent on the indication, disease process, and outcome of the therapy in children when compared to adults [1]. In most of the cases, scaling of the pharmacokinetic data from adults to children is desirable to either define the efficacious dose or for safety purposes to predict the range of drug exposure after administration of the selected dose(s). Risks associated with lack of efficacy or safety should be evaluated for every age category investigated and should ensure there is a sufficient safety margin across the predicted range of exposures.

Commonly used approaches for predicting exposures from adults to children are physiologically-based pharmacokinetic modeling (PBPK) and allometric scaling (AS) in combination with maturation of clearance for early life. PBPK models are system-specific models representing the physiology of the human body by means of mathematical equations and physiological parameters taking into account physiological blood flows, organ volumes, and partitioning between blood and organs. When scaling from adults to children using PBPK, acknowledged differences in all anatomical and physiological parameters are considered, being ontogeny on specific intrinsic clearances normalized to dry tissue weight of the eliminating organ [2–4]. Thereby variation in plasma concentration–time curves can in principle be predicted at various ages. Such predictions can be performed using commercially available software such as GastroPlus^®^, PKSim^®^ and SimCyp^®^. On the other hand, AS is a simplified scaling approach which allows prediction of volume of distribution and clearance using a power function for which the exponent is derived based on theoretical grounds linked to physiology [5]. Accordingly, total systemic clearance can be predicted by basically considering a 0.75-exponential relationship with weight. Besides growth, the gradual development of clearance pathways can be described with one single maturation function [6] which is established based on the large amount of pharmacokinetic data in children across a wide age-range, necessarily including very young ages [7].

Currently, AS maturation functions are often assumed to solely represent the ontogeny of the liver enzyme activity [8] and their use in the scaling of the maturation of other drugs implicitly assumes interchangeability with other approaches, as for example PBPK. In this investigation, this concept was challenged by evaluating the interchangeability of PBPK and AS in combination with maturation functions. To this end, this investigation provides information on the methodological uncertainty of clearance predictions, which can have a major influence on the estimation of the “first dose in children” and consequently on its risk benefit ratio. In addition, we aimed to provide insights into the physiological meaning of the AS maturation functions for which PBPK deemed suitable as it separately accounts for different physiological process including maturation of enzyme activity. A better understanding of the AS maturation functions can add in reducing the methodological uncertainty in future estimations of the “first dose in children”.

This investigation focused on the AS maturation functions for paracetamol [9] and morphine [10]. Paracetamol is mainly eliminated by glucuronidation (UGT1A6) and to a lesser extent by sulfation [11], while morphine is mainly metabolized by glucuronidation (UGT2B7) [12]. In addition, differences in the extraction ratio of paracetamol and morphine allowed investigation into its impact on the physiological meaning of the AS maturation function. Next, in order to evaluate the mechanisms affecting the interchangeability, hypothetical drugs with different pharmacokinetic properties and elimination via similar metabolic routes were used.

## Methods

### Morphine and paracetamol

PBPK models for morphine and paracetamol published earlier by Edginton et al. [2] and Willmann et al. [13] were used to simulate the expected population pharmacokinetics in children taking into account inter-individual variability. Morphine and paracetamol pharmacokinetics in 29 age groups (0, 3, 7 and 14 days; 1, 2, 3, 6, 9 months; 1, 1.5, and 2 to 18 years in incremental steps of 1 year) were simulated 200 times each. Pre-term neonates were not included in the simulations. In the simulations a dose of 10 mg morphine or 1,000 mg paracetamol was intravenously administered with an infusion time of 1 h. Simulated concentrations over a 24 h period were sufficiently long to ensure adequate clearance calculations in all age groups based on the non-compartmental *AUC* approach. From these individual simulations, quantiles were calculated and compared with the quantiles calculated from the simulations using the population pharmacokinetic models. The latter simulations were based on reported variances and log-normal distribution. In both simulations, the same set of demographics was used and correlation between demographics was accounted for. Demographics were compiled from the International Commission on Radiological Protection (ICRP) publication [14].

Clearance predictions using PBPK were then compared to clearance values obtained on basis of published population pharmacokinetic models, which were used to establish the AS maturation functions for paracetamol and morphine [9, 10].The paracetamol data set included children from 37 weeks to 14 years and the morphine dataset included children from 0 to 3 years, including pre-term and term neonates. For morphine both the model published by Bouwmeester et al. [15] and the descriptive model published by Knibbe et al. [16] were used for the comparisons. Both models were developed using the same data set in children from term-neonates to 3 years old, but the model published by Knibbe et al. [16] also included pre-term neonates. The AS maturation functions were graphically compared with the maturation functions for the enzyme activities and renal function as applied in the PBPK models.

### Simulations on hypothetical drugs

In total 108 hypothetical drugs were created with different pharmacokinetic properties being metabolized by either UGT2B7 (100%) or by a combination of UGT1A6 (65%) and sulfation (35%) as specified in Table 1. The physico-chemical and pharmacokinetic properties were pre-selected based on factors known to impact the extraction ratio of a compound. These properties were: lipophilicity (log P), percent relationship between clearance and liver blood flow, percent of maximum diffusion of the drug into the liver, and plasma protein binding. In order to reach pre-defined target values for these properties molecular weight and intrinsic clearance were adapted. All hypothetical drugs were assumed to bind to albumin. Hypothetical drugs with molecular weight greater than 1,000 Da and smaller than 90 Da were excluded from the analysis to assure that the created drugs were realistic small molecules.

**Table 1:** Overview of the pharmacokinetic properties of all the hypothetical drugs, paracetamol and morphine

Drug	Metabolic route^a^	Lipophilicity (log P)^a^	CL blood flow (%)^a^	Max. CL liver diffusion (%)^a^	Free fraction^a^	Intrinsic clearance (L/min)^b^	Molecular weight (g/moL)^b^	Extraction ratio^c^	*N*
Paracetamol	UGT1A6 (58%)	0.46	21	0	0.82	0.455	151.2	0.27	
	Sulfation (29%)								
	CYP2E1 (10%)								
	Renal (3%)								
Hypotheticals	UGT1A6 (65%)	0.46	10, 50, 90	10, 50, 90	0.05, 0.50, 0.95	0.17–164	96–865	0.0056–0.91	17
	Sulfation (35%)								
		1	10, 50, 90	10, 50, 90	0.05, 0.50, 0.95	0.17–164	185–600	0.017–0.89	14
		2	50, 90	10	0.05, 0.50, 0.95	2.40–171	370–667	0.074–0.72	6
Morphine	UGT2B7 (90.5%)	0.89	96	78	0.75	89.4	285.3	0.51	
	CYP3A4 (5%)								
	Renal (4.5%)								
Hypotheticals	UGT2B7	0.89	10, 50, 90	10, 50, 90	0.05, 0.50, 0.95	0.17–164	144–446	0.055–0.90	15

^a^Pre-defined pharmacokinetic properties

^b^Parameters adapted in the model

^c^Resultant pharmacokinetic property

The remaining hypothetical drugs were used to predict the clearance in children using both PBPK and AS in combination with maturation function. In all PBPK simulations 1 mg of the hypothetical drug was administered by an intravenous infusion of 30 min. Simulated concentration–time profiles were sufficiently long to assure adequate clearance calculation. When AS in combination with maturation function was used for predictions, Eq. 1 was applied:

1 $$ CL_{children} = {CL_{adults} \cdot \left( {\frac{{Weight_{children} }}{{Weight_{adults} }}} \right)^{0.75} \cdot {\text{AS maturation}}} $$

where *CL*_*adults*_ is the typical value estimated for the adult population established using PBPK and AS maturation is the maturation function used in combination with AS. The AS maturation function is an E_max_ function, monotonically increasing from neonates to adults and varying between 0 and 1. Weight in adults was fixed to 70 kg and weight in children was used as a size parameter and its distribution was the same as used for the PBPK simulations. The AS maturation function used was dependent on the metabolic route of the hypothetical drugs, i.e. for the hypothetical drugs metabolized by UGT2B7, the AS maturation function established for morphine was used [10], while for the hypothetical drugs metabolized by UGT1A6/Sulfation, the AS maturation function established for paracetamol was used [9] (Eq. 2).

2 $$ \begin{gathered} MF_{paracetamol} = \frac{{PMA^{3.92} }}{{PMA^{3.92} \cdot 5 4. 2}} \hfill \\ MF_{morphine} = 1 - 0.885 \cdot \exp \left[ {\frac{ - (PCA - 27) \cdot \ln (2)}{26.6}} \right] \hfill \\ \end{gathered} $$

where MF is maturation function, PMA is the post-menstrual age in weeks and PCA is post-conception age weeks.

### Graphical analysis

Graphical analyses were performed by assessing absolute clearance values or the ratio of the estimated clearances using AS in combination with the maturation function to the predicted clearances using PBPK versus age. Comparison of inter-individual variability was performed using a relative prediction interval calculated for every simulated age group according to Eq. (3)

3 $$ \begin{gathered} {Relative\; prediction\; interval}_{upper} = \frac{{95^{th} {Percentile CL} - 50^{th} {Percentile CL}}}{{50^{th} {Percentile}{CL}}} \hfill \\ {Relative\; prediction\; interval}_{lower} = \frac{{5^{th} {Percentile CL} - 50^{th} {Percentile CL}}}{{50^{th} {Percentile CL}}}. \hfill \\ \end{gathered} $$

The maturation functions were compared by plotting the ratio of the AS maturation function to the maturation function of enzyme activity and renal function as applied in the PBPK models. These ratios and the ratio of the clearance predictions using the two scaling approaches were plotted versus age. In all graphs, the logarithmic scale of the x axis was used to allow better visualization of the younger age groups.

### Software

PKSim^®^ version 4.2.4 (Bayer Technology Services GmbH, Leverkusen, Germany) in combination with RtoMoBi package version 1.3 (Bayer Technology Services GmbH, Leverkusen, Germany) was used to generate the models for the hypothetical drugs [17]. The software database on physiological information and clearance scaling was used to describe age-dependent differences in organ sizes, tissue composition (with respect to fat, protein and water content), blood flow rates, intrinsic clearances, and plasma protein abundance [2, 18, 19]. Paediatric simulations were performed using the population wrapper in MoBi^®^ version 2.3.4 (Bayer Technology Services GmbH, Leverkusen, Germany). R version 2.11.1 (R Foundation for Statistical Computing, Vienna, Austria) was used for AS simulations, including inter-individual variability, and graphical analysis.

## Results

### Morphine and paracetamol

**Fig. 1:**
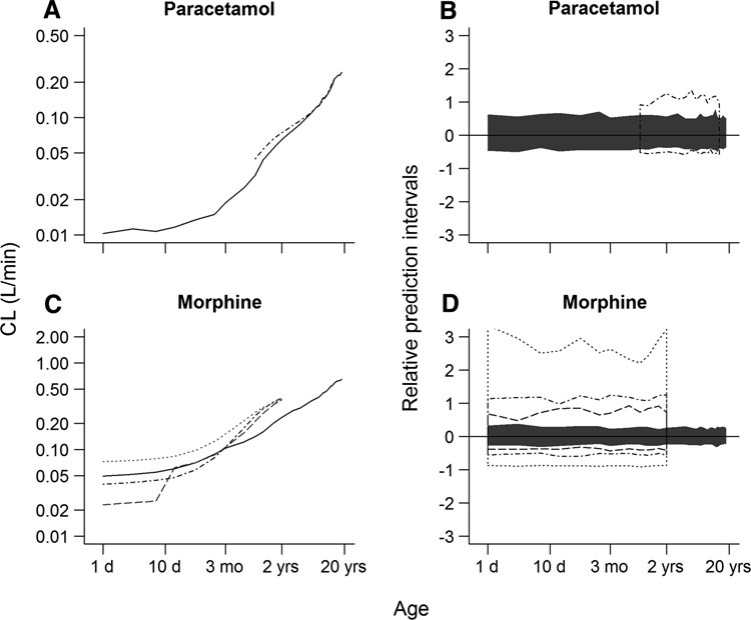
Comparison of absolute clearance values (**a** and **c**) and relative prediction intervals of the inter-individual variability (**b** and **d**) estimated using population pharmacokinetic models and predicted using PBPK models. *Solid black lines* and *intervals* represent the PBPK predictions and *dot-dashed black lines* and *intervals* represent the estimates using the population PK models reported by Anderson et al. [9] in **a** and **b** and Anand et al. [10] in **c** and **d**. The *dashed* *black lines* and *intervals* represent the estimates using the population PK models reported by Knibbe et al. [16] and the *dotted black lines* and *intervals* the estimates using the population PK models reported by Bouwmeester et al. [15]

Figure 1a and c compares the estimated clearances using population pharmacokinetic models and predicted clearances using PBPK for paracetamol and morphine versus age. For paracetamol (a), the clearance predictions using PBPK were in agreement with the estimated clearances for the age range of 1–18 years. For morphine (c), the PBPK predictions of clearance were slightly biased, with clearance being under-predicted in older children (>2.5 months) and over-predicted in younger children (<2.5 months). The estimated clearances by Bouwmeester et al. [15] were consistently higher but following the same pattern as the PBPK predictions. The relative prediction intervals of the inter-individual variability as estimated by the population pharmacokinetic models and as predicted by PBPK (Fig. 1b, d) showed that PBPK under-predicts the variability in all cases. In addition, large differences were observed in the relative prediction intervals between the population pharmacokinetic models described by Bouwmeester et al. [15] and Knibbe et al. [16] which were developed using the same data set from term neonates to infants of 3 years old. The simulations of inter-individual variability based on the model published by Bouwmeester et al. [15] are likely to be over-estimated as potential correlations in the omega covariance matrix of random inter-individual variability could not be considered in the simulations (not reported in the original publication).

**Fig. 2:**
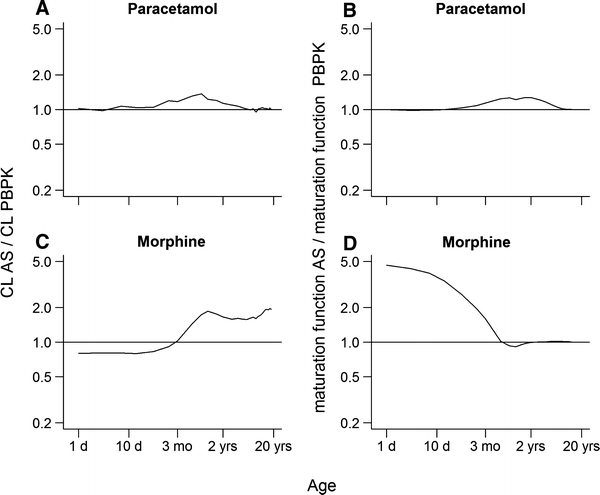
Ratio of the clearances estimated using population pharmacokinetic models to the clearances predicted using PBPK models (**a** and **c**) and the ratio of AS maturation functions to maturation functions of enzyme activities/renal function as applied in the PBPK models (**b** and **d**)

**Fig. 3:**
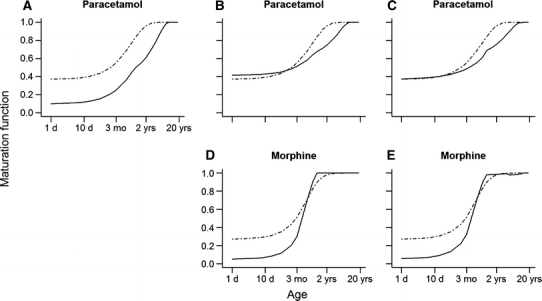
Comparison of the AS maturation functions with the maturation functions of different subset of enzymes activities involved in the metabolism of paracetamol (**a**, **b** and **c**) and morphine (**d** and **e**) as applied by the PBPK models. The *black* *dot*-*dashed* *lines* represent the AS maturation functions and the *black solid lines* represent the maturation function as applied in the PBPK models. In **a**, the *black solid line* represents the enzyme activities of UGT1A6 only; in **b**, the *black solid line* represents the combined enzyme activity of UGT1A6 and sulfation in a ratio of 65 to 35; in **c**, the *black solid line* represents the combined activity of all enzymes/routes including CYP2E1 and renal clearance; in **d**, the *black solid line* represents the enzyme activities of UGT2B7 only; and in **e**, the *black solid line* represents the combined activity of all enzymes/routes including CYP3A4 and renal clearance

The ratio of estimated clearance using population pharmacokinetic models to predicted clearance using PBPK models was evaluated in children from 0 to 18 years (Fig. 2a, c). Therefore, the estimated clearances and AS maturation functions had to be extrapolated beyond the age range investigated in the population pharmacokinetic models. For morphine, extrapolation of the AS maturation functions was applied to children older than 3 years and was considered acceptable as, at this age, maturation is already at adult levels (Fig. 3d, e). On the contrary, for paracetamol, extrapolation of the AS maturation function was also applied to children younger than 37 weeks where maturation is playing a significant role in the prediction of the total clearance (Fig. 3a–c). In this case, extrapolation was considered acceptable as it was in close agreement with the PBPK maturation function of the enzyme activity (Fig. 2b).

The calculated clearance ratios were used for direct comparisons with the ratios of the AS maturation function established using the population pharmacokinetic models to the maturation functions of the enzyme activities/renal function as applied in the PBPK models (Fig. 2). For paracetamol, comparison of both plots shows that the shape of the curves for the clearance ratio is similar to the shape of the curves for the maturation function (Fig. 2a, b). For morphine, the shape of the curves was not similar (Fig. 2c, d). Paracetamol and morphine maturation were also analyzed for the different metabolic pathways (Fig. 3). For paracetamol, the comparison of the AS maturation functions with the maturation functions in PBPK showed close agreement only when both maturation of UGT1A6 and sulfation in a ratio of 65 to 35 were considered (Fig. 3b). Inclusion of a maturation function for CYP2E1 and renal clearance did not result in a better agreement between the AS maturation function and the maturation function as applied in the PBPK model (Fig. 3c). Comparisons for morphine did not result in total agreement between AS maturation function and the maturation of UGT2B7 (Fig. 3d). Considering maturation of the enzyme activity of CYP3A4 and glomerular function did not result in a clear change of the shape of the resultant maturation function (Fig. 3e).

### Simulations on hypothetical drugs

**Fig. 4:**
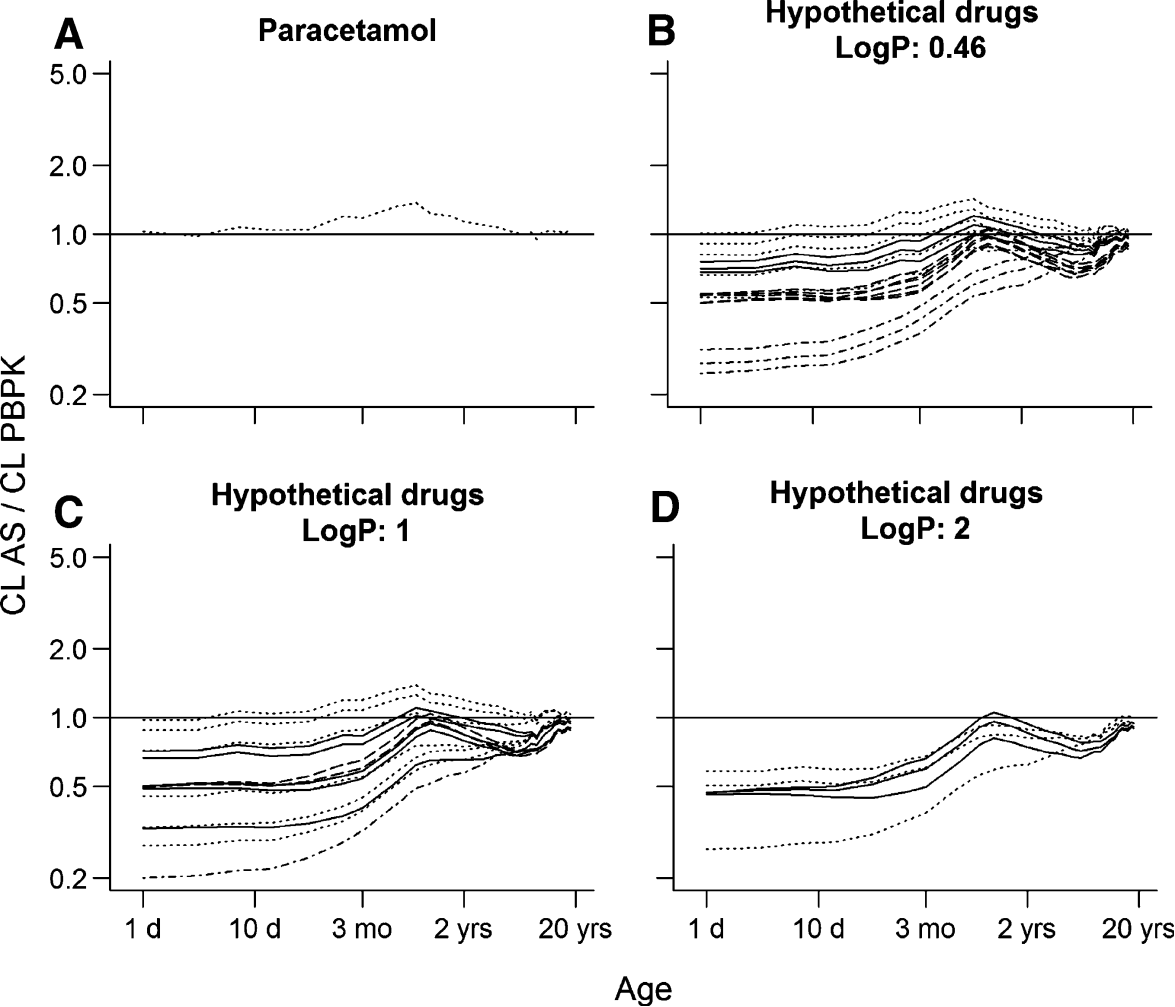
Ratio of clearance predictions using AS in combination with maturation function to clearance predictions using PBPK models (to be used as reference for the simulations using the hypothetical drugs). **a** represents the ratio of estimated clearance for paracetamol to predicted clearance by PBPK. The remaining represent the ratios for the hypothetical drugs with log P equal to 0.46 (**b**), 1 (**c**) and 2 (**d**). Each *line* represents one hypothetical drug: *dot-dashed black lines* represent drugs with very low extraction ratio (<0.05); *dotted black lines* represent drugs with low extraction ratio (0.05–0.3); *solid black lines* represent drugs with intermediate extraction ratio (0.3–0.7); and *dashed black lines* represent drugs with high extraction ratio (>0.7)

**Fig. 5:**
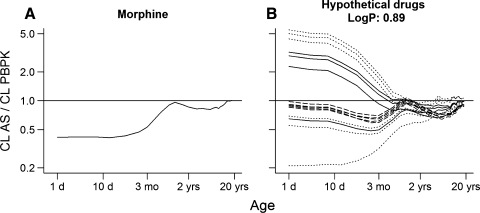
Ratio of clearance predictions using AS in combination with maturation function to clearance predictions using PBPK models (corrected for differences in clearances of adult values to allow its use as a reference for the simulations using the hypothetical drugs). **a** represents the ratio of estimated clearance for morphine to predicted clearance by PBPK. **b** represents the ratios for the hypothetical drugs with log P equal to 0.89. Each *line* represents one hypothetical drug: *dotted black lines* represent drugs with low extraction ratio (0.05–0.3); *solid black lines* represent drugs with intermediate extraction ratio (0.3–0.7); and *dashed black lines* represent drugs with high extraction ratio (>0.7)

In total 52 hypothetical drugs metabolized by either UGT1A6 and sulfation or UGT2B7 were used to predict the clearance in children by using AS in combination with a maturation function and by using PBPK models (Table 1). Figures 4b–d and 5b show the ratio of clearance predictions for hypothetical drugs with different pharmacokinetic properties and similar metabolic route as paracetamol and morphine, respectively. For all hypothetical drugs considerable differences in the prediction were mainly observed in children younger than approximately 1 year old. For older children the predictions using AS in combination with a maturation function and PBPK were in close agreement.

For hypothetical drugs with similar lipophilicity (log P), metabolic route and extraction ratio as paracetamol, the prediction ratios along the various ages (Fig. 4b) were in close agreement with those observed when the PBPK predictability was assessed for paracetamol (Fig. 4a). In addition, the magnitude of the prediction differences increased by increasing the extraction ratio of the hypothetical drug (Fig. 4b). On the other hand, hypothetical drugs with a very low extraction ratio (<0.05) showed the largest differences in the predictions (Fig. 4b). For hypothetical drugs with increased lipophilicity, similar metabolic route and similar extraction ratio as paracetamol (Fig. 4c, d), the prediction ratios between various ages were seldom comparable to the ones observed when the PBPK predictability was assessed for paracetamol (Fig. 4a). In addition, the prediction ratios no longer varied with the extraction ratio of the hypothetical drugs. Figure 5 shows that hypothetical drugs with similar lipophilicity, metabolic route and extraction ratio as morphine (b) do not always have similar prediction ratios as those used to assess the PBPK predictability for morphine (a).

## Discussion

The AS maturation function for clearance pathways in early life is assumed to solely represent the ontogeny of liver enzyme activity when scaling clearance of new pharmaceuticals entities [6, 8]. This approach is accepted by the regulatory authorities without the requirement for additional scaling to assess the uncertainty of the predictions. To this end, interchangeability with other also scientifically accepted approaches, such as PBPK, is implicitly assumed. In this investigation, we successfully challenged this assumption by comparing clearance predictions when using AS in combination with maturation function and PBPK for different hypothetical drugs. It should be stressed in this respect that the PBPK approach separately accounts for drug properties and for ontogeny of different physiological and anatomical aspects, while the AS approach account for size changes and adopts a single maturation function for all compounds with a similar metabolic route. The characteristics of the PBPK approach allowed us also to gain insights into the physiological meaning of the AS maturation function and thereby challenge the assumption that it solely represents enzyme activity.

In the first step of this investigation, comparison between PBPK predictions and population pharmacokinetic models describing pediatric clinical data were ultimately meant to challenge the assumption that AS maturation functions solely represent maturation of liver enzyme activity. Paracetamol and morphine were used as paradigm drugs based on the availability of AS maturation functions and were deemed interesting because of the differences in extraction ratio. For paracetamol, PBPK predicted and estimated clearances were in good agreement (Fig. 1a), while for morphine, the small differences observed in young children, were randomly distributed around the PBPK predictions (Fig. 1c). The differences in estimated clearance could be caused by study differences in the data used for model development and/or structure of the model applied [10, 15, 16]. Comparison between PBPK predicted and estimated inter-individual variability of clearance indicates that PBPK under-predicts the inter-individual variability (Fig. 1b, d). Potential under-prediction of inter-individual variability is most likely to be caused by the fact that variability in the anatomical and physiological parameters has not yet been well established. On the other hand, increased inter-individual variability estimated by population pharmacokinetic models of morphine developed by Anand et al. and Bouwmeester et al. suggests that fixing AS exponents into the model may result in an over-estimation of the inter-individual variability compensating for differences between “real” and fixed exponent. None of these results should not be interpreted to suggest that one approach can be preferred above the other, but to provide a basis for a better understanding of the AS maturation function.

For this reason, clearances and maturation functions for paracetamol and morphine were then compared with PBPK clearance prediction and maturation function in children from term neonates to 18 years. For paracetamol, a similar pattern was observed for the ratio of clearances and maturation functions, suggesting that the AS maturation function fully represents the PBPK maturation of the enzyme activity (Fig. 2a, b). On the other hand, lack of similarity observed for morphine (i.e., patterns show different directions and magnitude) indicates that AS maturation function represents more than only the maturation of the enzyme activity (Fig. 2c, d). This could be explained by differences in extraction ratio between paracetamol and morphine. Paracetamol is a low extraction ratio drug for which ontogeny of enzyme activity plays a main role in the maturation of the total clearance, while morphine is an intermediate extraction ratio drug for which ontogeny of multiple physiological process such as for example liver blood flow are also expected to play a role in the maturation of the total clearance [20]. In addition, AS maturation function for morphine potentially comprises ontogeny of active processes determining the pharmacokinetics of morphine [21]. Furthermore, this first part of the investigation suggested that paracetamol is primarily metabolized by UGT1A6 and sulfation (65/35%) (Fig. 3a–c) and morphine mainly by UGT2B7 (Fig. 3d, e), being in agreement with the literature [11, 12].

In the second step of the investigation, it was examined to what extent the findings for paracetamol and morphine would also apply to hypothetical drugs sharing solely the same metabolic route. In other words, it was evaluated for which hypothetical drugs the clearance ratio predicted using PBPK and AS in combination with maturation function showed a similar pattern as observed for paracetamol and morphine. One could interpret this step of the investigation as a kind of sensitivity analysis that evaluates the impact of changes in compound properties on the prediction ratio of the total clearance in children. Hence, hypothetical drugs were created assuming similar metabolic routes as for paracetamol and morphine but with different pharmacokinetic properties pre-selected to lead to different extraction ratios (Table 1). The hypothetical drugs were used to predict the clearance in children by means of PBPK and AS in combination with maturation function.

For all hypothetical drugs, the clearance predictions using PBPK and AS in combination with maturation function provided nearly identical results in children older than 1 year, which is in agreement with previous findings [19]. However, interchangeability in children younger than 1 year for hypothetical drugs having similar metabolic routes as paracetamol was only observed for drugs with similar extraction ratio and lipophilicity (log P) (Fig. 4). These compounds differed from paracetamol only in intrinsic clearance. From a mechanistic point of view, the extraction ratio and lipophilicity determine the amount of drug that is available in the liver for metabolism. Interestingly, for the hypothetical drugs sharing similar metabolic route as morphine, considering extraction ratio and lipophilicity were shown not to be sufficient to guarantee the same pattern in clearance prediction ratio as observed for morphine in children younger than 1 year (Fig. 5). This could be attributed to the fact that the ontogeny of transporters was not considered in the PBPK simulations while it was likely to be part of the maturation function used in the AS simulations [21].

Altogether, these findings show that interchangeability in clearance predictions using PBPK and AS in combination with maturation function cannot be assumed in children younger than 1 year for the metabolic routes investigated. Not surprisingly, as at young ages, the simultaneous ontogeny of multiple physiological processes is likely to influence the total clearance of a drug [20]. Also for this reason, estimation of one single AS maturation function probably aggregates ontogeny of not only enzyme activity but of other physiological process. Therefore, extrapolation of AS maturation function between compounds is most likely only acceptable in very specific circumstances, thereby restricting its use for scaling from adults to children and consequently restricting its applicability in paediatric drug development.

In conclusion, this investigation provided insight into the physiological meaning of the AS maturation functions. Moreover, it showed that it is incorrect to assume that PBPK and AS in combination with maturation function will always be interchangeable. For this reason, methodological uncertainty should be considered on a case-by-case basis when estimating the “first dose in children” and assessing its risk and benefits. In order to reduce this methodological uncertainty the obtained insights on the physiological meaning of the AS maturation function should be acknowledged in future scaling. Further, extensive validation of different prediction approaches using pediatric clinical data is required to allow improvement of the predictability and enhance assessment of the uncertainties of both approaches.

## References

[1] European Medicines Agency (2007) ICH Topic E11 clinical investigation on medicinal products in the paediatric population. CPMP/ICH/2711/99. 2007

[2] Edginton AN, Schmitt W, Willmann S (2006). Development and evaluation of a generic physiologically based pharmacokinetic model for children. Clin Pharmacokinet.

[3] Nestorov I (2003). Whole body pharmacokinetic models. Clin Pharmacokinet.

[4] Johnson TN, Rostami-Hodjegan A, Tucker GT (2006). Prediction of the clearance of eleven drugs and associated variability in neonates, infants and children. Clin Pharmacokinet.

[5] West GB, Brown JH, Enquist BJ (1997). A general model for the origin of allometric scaling laws in biology. Science.

[6] Anderson BJ, Holford NH (2008). Mechanism-based concepts of size and maturity in pharmacokinetics. Annu Rev Pharmacol Toxicol.

[7] Bouillon-Pichault M, Jullien V, Bazzoli C, Pons G, Tod M (2011). Pharmacokinetic design optimization in children and estimation of maturation parameters: example of cytochrome P450 3A4. J Pharmacokinet Pharmacodyn.

[8] Tod M, Jullien V, Pons G (2008). Facilitation of drug evaluation in children by population methods and modelling. Clin Pharmacokinet.

[9] Anderson BJ, Pons G, Autret-Leca E, Allegaert K, Boccard E (2005). Pediatric intravenous paracetamol (propacetamol) pharmacokinetics: a population analysis. Paediatr Anaesth.

[10] Anand KJ, Anderson BJ, Holford NH, Hall RW, Young T, Shephard B, Desai NS, Barton BA (2008). Morphine pharmacokinetics and pharmacodynamics in preterm and term neonates: secondary results from the NEOPAIN trial. Br J Anaesth.

[11] Zhao L, Pickering G (2011). Paracetamol metabolism and related genetic differences. Drug Metab Rev.

[12] Glare PA, Walsh TD (1991). Clinical pharmacokinetics of morphine. Ther Drug Monit.

[13] Willmann S, Edginton AN, Coboeken K, Ahr G, Lippert J (2009). Risk to the breast-fed neonate from codeine treatment to the mother: a quantitative mechanistic modeling study. Clin Pharmacol Ther.

[14] International Commission on Radiological Protection (ICRP) (2002) Basic anatomical and physiological data for use in radiological protection: reference values. ICRP publication 89. Elsevier Science, Amsterdam

[15] Bouwmeester NJ, Anderson BJ, Tibboel D, Holford NH (2004). Developmental pharmacokinetics of morphine and its metabolites in neonates, infants and young children. Br J Anaesth.

[16] Knibbe CA, Krekels EH, van den Anker JN, DeJongh J, Santen GW, van DM, Simons SH, van Lingen RA, Jacqz-Aigrain EM, Danhof M, Tibboel D (2009). Morphine glucuronidation in preterm neonates, infants and children younger than 3 years. Clin Pharmacokinet.

[17] Eissing T, Kuepfer L, Becker C, Block M, Coboeken K, Gaub T, Goerlitz L, Jaeger J, Loosen R, Ludewig B, Meyer M, Niederalt C, Sevestre M, Siegmund HU, Solodenko J, Thelen K, Telle U, Weiss W, Wendl T, Willmann S, Lippert J (2011). A computational systems biology software platform for multiscale modeling and simulation: integrating whole-body physiology, disease biology, and molecular reaction networks. Front Physiol.

[18] Edginton AN, Schmitt W, Voith B, Willmann S (2006). A mechanistic approach for the scaling of clearance in children. Clin Pharmacokinet.

[19] Edginton AN, Willmann S (2006). Physiology-based versus allometric scaling of clearance in children; an eliminating process based comparison. Paediatr Perinat Drug Ther.

[20] Pang KS, Rowland M (1977). Hepatic clearance of drugs. I. Theoretical considerations of a “well-stirred” model and a “parallel tube” model. Influence of hepatic blood flow, plasma and blood cell binding, and the hepatocellular enzymatic activity on hepatic drug clearance. J Pharmacokinet Biopharm.

[21] Mashayekhi SO, Sattari MR, Routledge PA (2010). Evidence of active transport involvement in morphine transport via MDCKII and MDCK-PGP cell lines. Res Pharm Sci.

